# Altered locomotion and anxiety after exposure to SiO_2_ nanoparticles in larval zebrafish

**DOI:** 10.1038/s41598-025-02599-3

**Published:** 2025-05-25

**Authors:** Pauline Y Shen, Jiaze Wu, Gavin Pu, Kai Huang, Qian Lin

**Affiliations:** 1 Department of Cell and Systems Biology, University of Toronto, Toronto, ON M5S 3G5 Canada; 2 Department of Materials Science and Engineering, University of Toronto, Toronto, ON M5S 3E4 Canada

**Keywords:** Neuroscience, Toxicology, Nanotoxicology

## Abstract

Nanoparticles (NP) have been driving the rapid advancement of nanomedicines in recent decades. However, their wide application also raises safety concerns, particularly their neurotoxicity due to their ability to cross the blood-brain barrier and accumulate in the brain, which remains largely underexplored. Here, we used silica nanoparticles (SiO_2_ NP) as a model to study the neurotoxicity of nanomedicine, based on their general features and functionalities. Using the light/dark preference behavioral assays of larval zebrafish, we focused on the neurotoxic consequences of exposure to an array of low concentrations of SiO_2_ NP, which reflected real-world conditions compared to previous studies, and examined the effect of different exposure durations. We observed dose-dependent and temporally sensitive changes in locomotor activities and elevated anxiety-related behaviors after exposure. Strikingly, exposed animals exhibited biphasic alteration: hypo-locomotion after 24-hour exposure and hyper-locomotion after 48-hour exposure. Our work provided real-world relevant behavioral insights, and highlighted the biphasic response and the temporal sensitivity of the SiO_2_ NP neurotoxicity. These findings underscore the potential neurotoxic risks of nanomedicine applications and emphasize the urgent need for further research into NP-associated neurotoxicity and public awareness.

**Supplementary Information:**

The online version contains supplementary material available at 10.1038/s41598-025-02599-3.

## Introduction

The integration of nanotechnologies into conventional medical applications, namely nanomedicine, has advanced rapidly in recent decades^1-4^. This field uses nanoparticles (NPs), ranging from 1 to 100 nm in size, to enhance target specificity, drug bioavailability, therapeutic window, and treatment efficacy of conventional approaches^2,5-8^. These NPs approximate the scale of biological molecules, facilitating improved interaction at the cellular level^9,10^. The pandemic has demonstrated potential roles of nanomedicine, as evidenced by the worldwide administration of mRNA-loaded nanoparticle COVID-19 vaccines to combat the virus^11^. As of June 30, 2024, over 107 million vaccine doses have been administered across Canada^12^, accounting for 63,000 g of NPs (assuming 0.59 mg mass of pure NPs per dose)^13^. However, despite the successful applications of nanomedicines, their ability to cross the blood-brain barrier (BBB) raises significant concerns about their safety, particularly their potential to cause neurotoxicity.

Silica nanoparticle (SiO_2_ NP) is an important model for studying NP behaviors in biological systems and is undergoing several clinical trials^7,14,15^. It holds capabilities in drug delivery and offers advantages in superior structural and physicochemical properties, including size, stability, structural tunability, surface functionality, loading capacity, reproducibility, or biocompatibility^16-22^. SiO_2_ NPs show promise for advanced biomedical applications such as optical imaging, cancer therapy, targeted drug delivery, or controlled gene and protein release^18,23-29^. Additionally, its synthetic simplicity and abundance on Earth make its manufacturing scalable and eco-friendly^21,22,30^. These traits enhance their applicability as a general-purpose nanotechnology, making SiO_2_ NP a prominent testbed for studying nanomedicine. Furthermore, SiO_2_ NPs have been extensively applied across various industries of diverse fields, ranging from mechanical polishing and food production to water treatment^23,24,31^. It is listed among the top five nanomaterials in nanotechnology consumer products^32^. In 2014, global production of SiO_2_ nanoparticles was reported to be substantial, ranging from 185,000 to 1.4 million tons, exceeding that of other nanoparticles^33^. The widespread application of SiO_2_ NPs emphasizes the importance of assessing their potential negative impacts on living organisms and nervous systems.

NPs can enter the human body through multiple routes, such as inhalation, ingestion, injection, and transdermal delivery^34^. They can bypass the blood-brain barrier (BBB) and reach the brain via blood circulation or olfactory routes through the respiratory tract based on previous in vivo (rats and medaka) and in vitro studies^4,35-38^. When administered intranasally in rats, SiO_2_ NPs accumulated in brain regions such as the striatum and the hippocampus^39^. In vitro, SiO_2_ NPs caused adverse functional effects on the brain by increasing oxidative stress and changing cytokine release, thus elevating the potential risk of neurodegenerative disorders^39,40^. These findings further emphasize the criticality of a deeper understanding of the neurotoxicity of SiO_2_ NP, which remains insufficiently explored.

Zebrafish offers a powerful model for neurotoxicology^41^. Their rapid development and high physiological and genetic homology to mammalian brains allow comparable toxicity profiles^41-45^. In parallel, their behavioral assays offer high-throughput, sensitive, and quantifiable indicators of neurological effect^42,46-49^. Larval fish’s small size and transparent brains make them particularly suitable for in vivo imaging with cellular and subcellular resolution^42,46,50^, enabling direct measurement of the effects of substances on neural tissues^51-53^. They have been widely used in large-scale or even whole-brain neural recordings to study neurodynamics^54-56^. So far, research on the neurotoxic effects of SiO_2_ NP in zebrafish predominantly focuses on the embryonic stage^57,58-60^, which inevitably introduces developmental disruptions and complicates dissecting neurotoxicity. Additionally, while previous studies predominantly used high concentrations of NPs, often exceeding 100mg/L^61–65^, many consumer aerosol-based products contain only 13 mg/L of SiO_2_ NPs^66^. However, studying neurotoxicants at high concentrations typically provides mechanistic rather than toxicological insights^64^. Thus, research employing concentrations higher than those typically observed in real-life scenarios may not accurately reflect actual toxicological risks^64,66^. Hence in this study, we focused on early detection of nanomaterial neurotoxicity using SiO_2_ NP with low-dose exposure; using larval zebrafish, we aim to provide a model for further investigation of neurological mechanisms.

The present study leverages these advantages of zebrafish larvae, by developing a neurobehavioral framework to assess the potential impacts of nanomaterial exposure. Using a light/dark preference behavioral assay, we investigated the neurotoxic effects of SiO_2_ NP and evaluated effects based on both concentration and exposure duration. Our findings demonstrated a complex, dose- and time-dependent relationship between SiO_2_ NP exposure and behavioral outcomes, particularly in locomotion, revealing a biphasic effect of the nanomaterials. These results contribute to a better understanding of the dynamic interactions between nanomaterials and neurobehavioral responses. Furthermore, our behavioral framework has the potential to support large-scale behavioral screening for testing the neurotoxicity of other NPs. Taken together, our findings indicate that SiO_2_ NPs alter the motor systems of zebrafish larvae and lays the groundwork for future neurotoxicity profiling of NPs.

## Result

### SiO_2_ NPs characterization and experimental setup

**Fig. 1:**
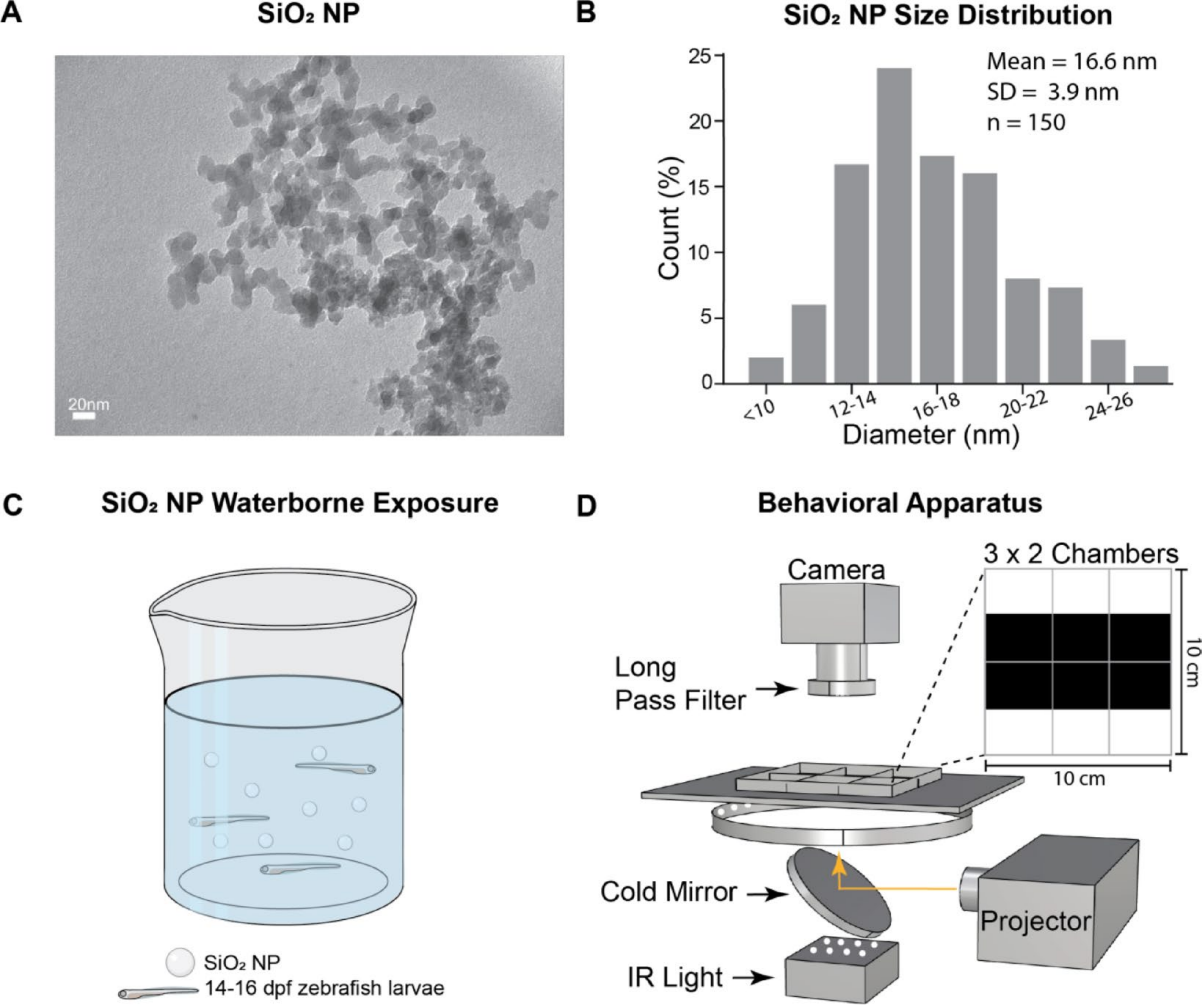
Silica nanoparticles (SiO_2_ NPs) size distribution and experimental set-up. (**A**) Transmission electron microscopy (TEM) image of SiO_2_ NPs. Scale bar, 20 nm. (**B**) Distribution of SiO_2_ NPs diameter sizes. (**C**) Schematic of the waterborne exposure method for SiO_2_ NPs. (**D**) Custom-built behavioral set-up for the light/dark preference assay. Fish were tested in a 3D-printed six-well chamber, with dark/light stimuli projected onto the bottom. Top-right, a top view of the chamber with light and dark stimuli for the assay. The orange arrow indicates the projection path of the visual stimuli.

To characterize SiO_2_ NPs, we used transmission electron microscopy (TEM) and found that they are primarily uniform in size but irregularly spherical in shape (Fig. 1A). The average particle size is quantified as 16.6 nm ± 3.90 nm (mean ± standard deviation) using ImageJ^67^, displaying a monomodal size distribution (Fig. 1B). Dynamic light scattering (DLS) characterizations were conducted with the Zetasizer (Nano ZS, Malvern Instruments Ltd., UK) to measure the hydrodynamic size and zeta-potential of the SiO_2_ NPs at a concentration of 100 mg/L in deionized water (Figure S1).

Fish at 14–15 days post-fertilization (dpf) were exposed to SiO_2_ NPs via waterborne exposure for either 24 hours (24h) or 48 hours (48h) at an array of concentrations (Fig. 1C). To assess the behavioral consequences of exposure, we adapted a classical light/dark preference assay to measure sensory function, anxiety level, and locomotion^68,69^. For this, we built a behavioral apparatus (Fig. 1D). Zebrafish were placed in a 3D-printed six-well chamber, one fish per well, where dark and light stimuli were projected onto the bottom, and their behavior was recorded 6 fish in parallel at 15 Hz for subsequent analysis (Fig. 1D), including phototaxis, anxiety-related behaviors, and locomotion.

### SiO_2_ NP exposure does not influence larval phototaxis

**Fig. 2:**
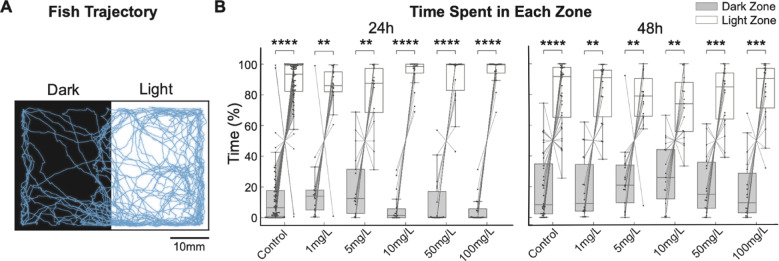
Consistent light preference among SiO_**2**_ NP-exposed larvae. (**A**) A representative fish trajectory demonstrates light preference in a dark/light preference assay. (**B**) Percentage of time spent in dark versus light side for larvae exposed to varying concentrations of SiO_2_ NP. Left panel, fish were exposed for 24 hours (24h), and all groups exhibited a significant preference for light. *n* = 70 for the control group and *n* = 17–18 for the experimental groups. ****p < 1E-9 for control (*n* = 70), **p = 0.0077 for 1 mg/L (*n* = 18), **p = 0.0023 for 5 mg/L (*n* = 17), ****p < 0.0001 for 10, 50, and 100 mg/L (*n* = 18 for each), Wilcoxon signed-rank test with Bonferroni correction. Right panel, fish were exposed for 48 hours (48h), and all groups exhibited a significant preference for light. *n* = 36 for the control group and *n* = 17–18 for the experimental groups. ****p < 1E − 6 for control (*n* = 36), **p = 0.0011 for 1 mg/L (*n* = 18), **p = 0.0081 for 5 mg/L (*n* = 17), **p = 0.0064 for 10 mg/L (*n* = 17), ***p = 0.0006 for 50 mg/L (*n* = 18), ***p = 0.0005 for 100 mg/L (*n* = 18), Wilcoxon signed-rank test with Bonferroni correction.

We first assessed whether the sensory process was altered by SiO_2_ NPs exposure, by measuring preference under dark and light visual stimuli. Larval trajectory showed a strong preference in the light zone, a robust positive phototaxis that was unaffected by SiO_2_ NPs exposure (Fig. 2A; Video S1). Quantitation of the time spent in each zone revealed that larvae across all control and experimental groups showed a significant preference for the light zone for both 24h and 48h (Fig. 2B, 24h: ****p < 1E-9 for control, **p < 0.01 for 1 and 5 mg/L, ****p < 0.0001 for 10, 50, and 100 mg/L; 48h: ****p < 1E − 6 for control, **p < 0.01 for 1, 5, and 10 mg/L, ***p = 0.001 for 50 and 100 mg/L. Wilcoxon signed-rank test with Bonferroni correction). This indicates that the larvae’s visual sensory functions and phototaxis remain unaffected by exposure to SiO_2_ NP.

### SiO_2_ NP elevates anxiety-related behaviors in larvae

**Fig. 3:**
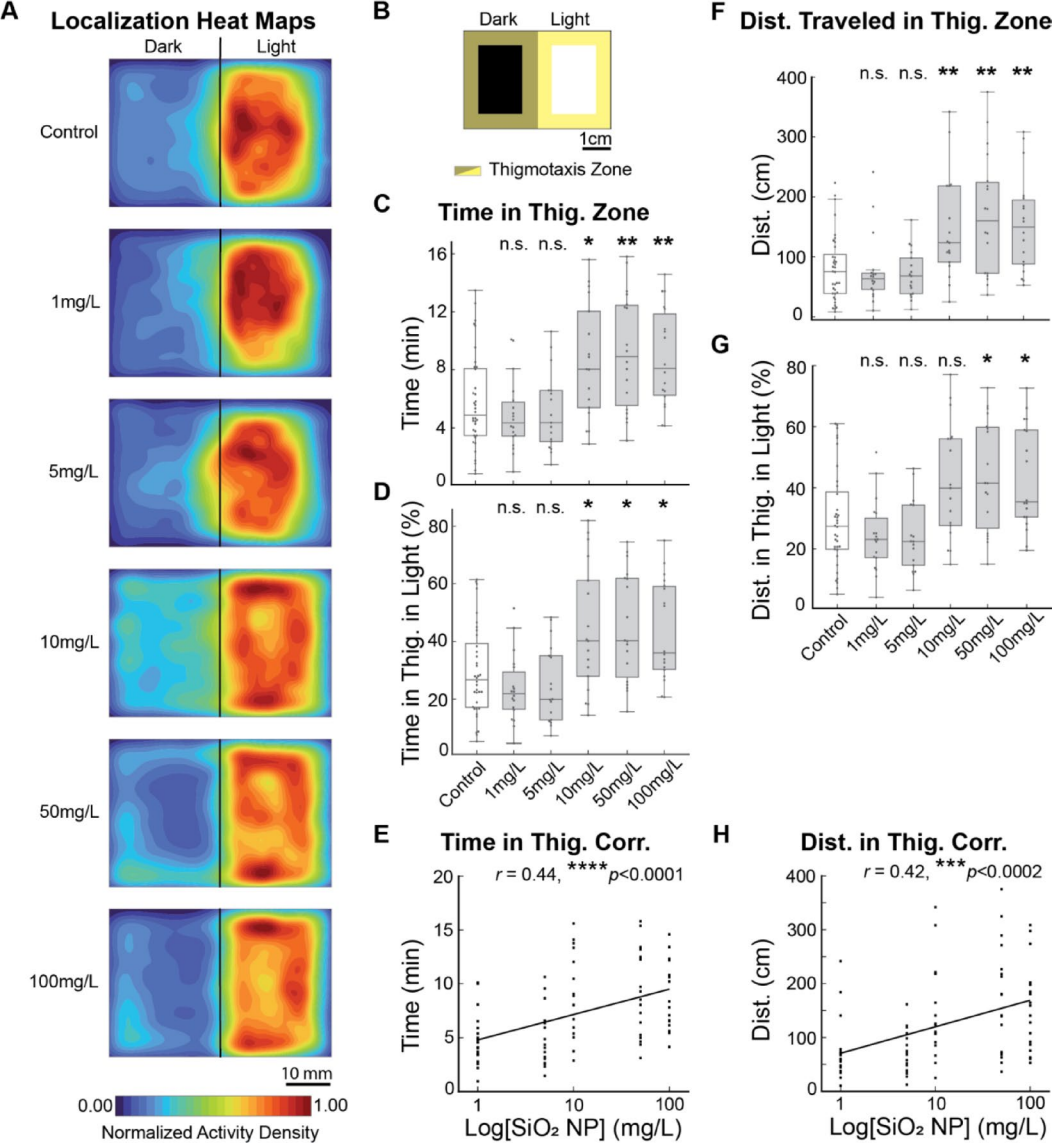
SiO_2_ NP elevates anxiety-related behaviors in larvae. (**A**) Heat maps of fish’s localization during light/dark preference behavioral assay post-48h exposure to SiO_2_ NPs. (**B**) The thigmotaxis zone is defined as areas within 5 mm from the chamber wall and the border of the dark and light sides. (**C**) SiO_2_ NP exposure alters the time spent in the thigmotaxis (Thig.) zone. All groups differ significantly, ****p < 0.0001, Kruskal-Wallis test. Compared to the control group, fish exposed to 10 mg/L and higher spent significantly longer times in the thigmotaxis zone. *p = 0.0265 fro 10 mg/L, **p = 0.0076 for 50 mg/L, **p = 0.0076 for 100 mg/L, one-tailed Mann-Whitney U test with Holm-Bonferroni correction. *n* = 36 for the control group and *n* = 17–18 for the experimental groups. (**D**) The percentage of time spent in the thigmotaxis zone on the light side differs significantly among all groups. ****p < 0.0001, Kruskal-Wallis test. Compared to the control group, fish exposed to 10 mg/L and higher spent significantly longer times in the thigmotaxis zone. *p = 0.032 for 10 mg/L, *p = 0.0102 for 50 mg/L, *p = 0.0122 for 100 mg/L, one-tailed Mann-Whitney U test with Holm-Bonferroni correction. (**E**) The time spent in the thigmotaxis zone positively correlates with exposure concentration. *r* = 0.44, ****p < 0.0001. (**F**) SiO_2_ NP exposure alters the distance traveled in the thigmotaxis (Thig.) zone. All groups differ significantly, ****p < 0.0001, Kruskal-Wallis test. Compared to the control group, fish exposed to 10 mg/L and higher traveled significantly longer distances. **p = 0.0055 for 10 mg/L, **p = 0.0031 for 50 mg/L, **p = 0.0023 for 100 mg/L, one-tailed Mann-Whitney U test with Holm-Bonferroni correction. (**G**) The percentage of distance traveled in the thigmotaxis zone on the light side differs significantly among all groups. ****p < 0.0001, Kruskal-Wallis test. Compared to the control group, fish exposed to 50 and 100 mg/L exhibited more activity in the thigmotaxis zone. *p = 0.0136 for 50 mg/L, *p = 0.0136 for 100 mg/L, one-tailed Mann-Whitney U test with Holm-Bonferroni correction. (**H**) The distance traveled in the thigmotaxis zone positively correlates with concentration. *r* = 0.42, ***p < 0.0002.

As NPs have been reported to induce anxiety^70-72^, we then assessed the anxiety-related behaviors. By visualizing larval exploration patterns within the arena using heat maps of their normalized activity density (see Methods), we observed increased thigmotaxis, where fish spent more time near the wall, in larvae exposed to higher concentrations of SiO_2_ NPs (Fig. 3A, 48h shown). In the defined thigmotaxis zone (Fig. 3B), larvae exposed to 10 mg/L and higher, spent significantly longer times than the control (Fig. 3C; ****p < 0.0001 across all the group, Kruskal-Wallis test; *p = 0.026 for 10 mg/L, **p < 0.01 for 50 and 100 mg/L, one-tailed Mann-Whitney U test with Holm-Bonferroni correction). Even in their preferred light side of the arena, these larvae also spent longer times in the thigmotaxis zone, implying an induced anxious state of the animal (Fig. 3D; ****p < 0.0001, Kruskal-Wallis test; *p < 0.05 for 10, 50, and 100 mg/L compared to the control, one-tailed Mann-Whitney U test with Holm-Bonferroni correction). Notably, the time spent in the thigmotaxis zone shows a positive correlation with the concentration, suggesting a higher tendency to induce anxiety as concentrations increase (Fig. 3E; *r* = 0.44, ****p < 0.0001).

Similarly, with regards to larval activities in the thigmotaxis zone, concentrations of 10 mg/L and higher groups traveled significantly longer distances than the control group (Fig. 3F; ****p < 0.0001, Kruskal-Wallis test; **p < 0.01 for 10, 50, and 100 mg/L, one-tailed Mann-Whitney U test with Holm-Bonferroni correction). In the thigmotaxis zone on the preferred light side, 50 and 100 mg/L groups exhibited significantly longer distances traveled than the control group (Fig. 3G; ****p < 0.0001, Kruskal-Wallis test; *p < 0.05 for 50 and 100 mg/L, one-tailed Mann-Whitney U test with Holm-Bonferroni correction). Notably, the distance traveled in the thigmotaxis also positively correlated with the concentration (Fig. 3H; *r* = 0.42, ***p < 0.0002). These collectively suggest that SiO_2_ NPs exposure elevates anxiety-related behaviors.

### SiO_2_ NP induces dose-dependent alterations in larval exploration patterns

**Fig. 4:**
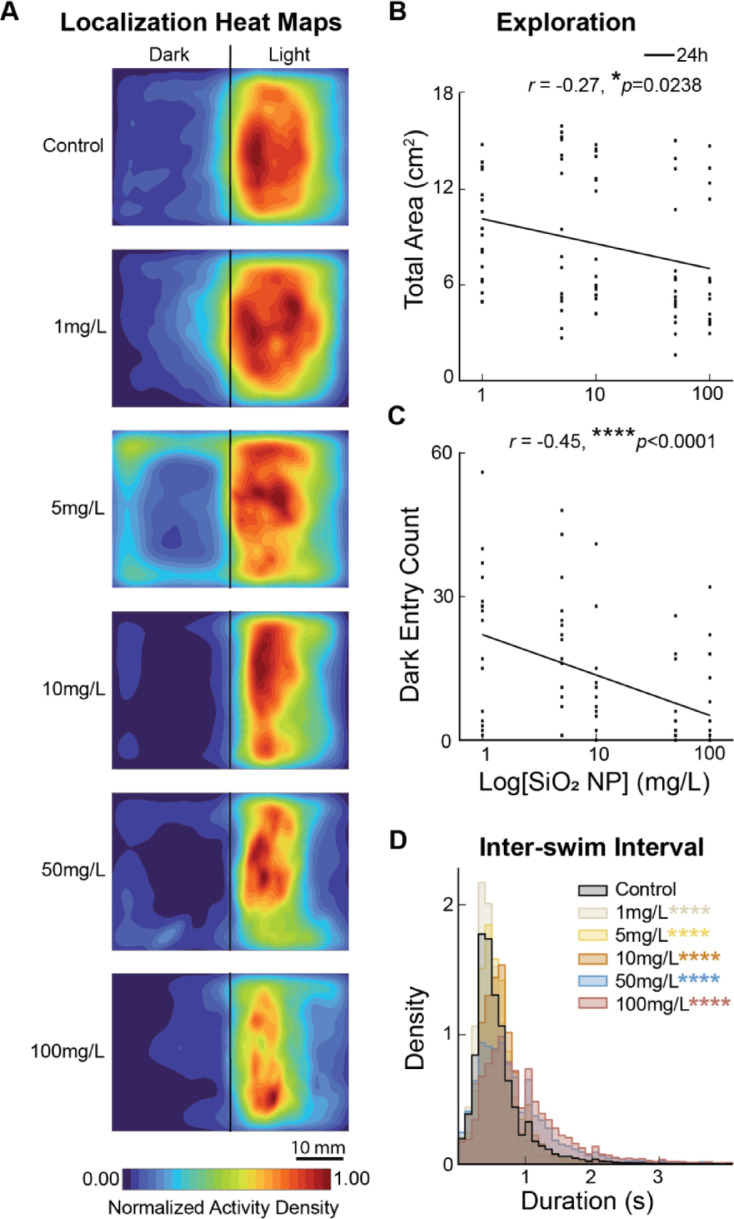
Alterations in exploration patterns and anxiety-related behaviors are dose-dependent. (**A**) Heat maps of fish’s localization during light/dark preference behavioral assay post-24h of exposure to SiO_2_ NPs. (**B**) The total area explored negatively correlates with concentration. *r*=-0.27, *p = 0.0238. (**C**) The number of entries into the dark side negatively correlated with concentration. *r*=-0.45, ****p < 0.0001. (**D**) Inter-swim interval distribution of 24h groups. Bins of 0.1s. Compared to the control group, larvae exposed to 1 mg/L exhibited a higher frequency of shorter intervals between swimming events, while those exposed to concentrations of 5 mg/L and higher showed rightward shifts in the interval distribution with longer inter-swim intervals. ****p < 1E-270 for 1 mg/L, ****p < 1E-88 for 5 mg/L, ****p < 1E-323 for 10 mg/, ****p < 1E-323 for 50 mg/L, ****p < 1E-323 for 100 mg/L, Kolmogorov–Smirnov test with Bonferroni correction.

Then we visualized the exploration patterns for the 24h SiO_2_ NP exposure groups using heat maps, and we observed a decrease in the area explored at higher concentrations (Fig. 4A; Video S1). The area explored was negatively correlated with the concentrations in both the area explored and the number of entries into the dark side (Fig. 4B, C; *r*=-0.27, *p = 0.0238; *r*=-0.25, *p = 0.0363 respectively). This means that higher concentrations could inhibit exploratory behavior. However, the time and distance traveled did not show significant differences (*p* > 0.05, Kruskal-Wallis test); we thus assessed the inter-swim interval (ISI), which represents the duration of time between two consecutive swimming events (see Methods). As expected, we observed a rightward shift in the distributions of ISI after exposure with concentrations > 5 mg/L. The ISI distribution peaked at approximately 0.5s for 5 mg/L and 0.7s for groups > 10 mg/L compared to the control at 0.4s (Fig. 4D; ****p < 1E-80 for 1, 5, 10, 50, and 100 mg/L, Kolmogorov–Smirnov test with Bonferroni correction). This suggests micro-scale locomotion alterations that affect larvae’s behavior continuum, specifically exploratory behavior suppression with less frequent dark side entries as concentration increases. Taken together, these changes in exploratory patterns suggest either an anxiogenic or sedative effect of SiO_2_ NP exposure, thus promoting their innate avoidance of dark environments and increasing ISI.

### SiO_2_ NP alters locomotor activities in larvae

**Fig. 5:**
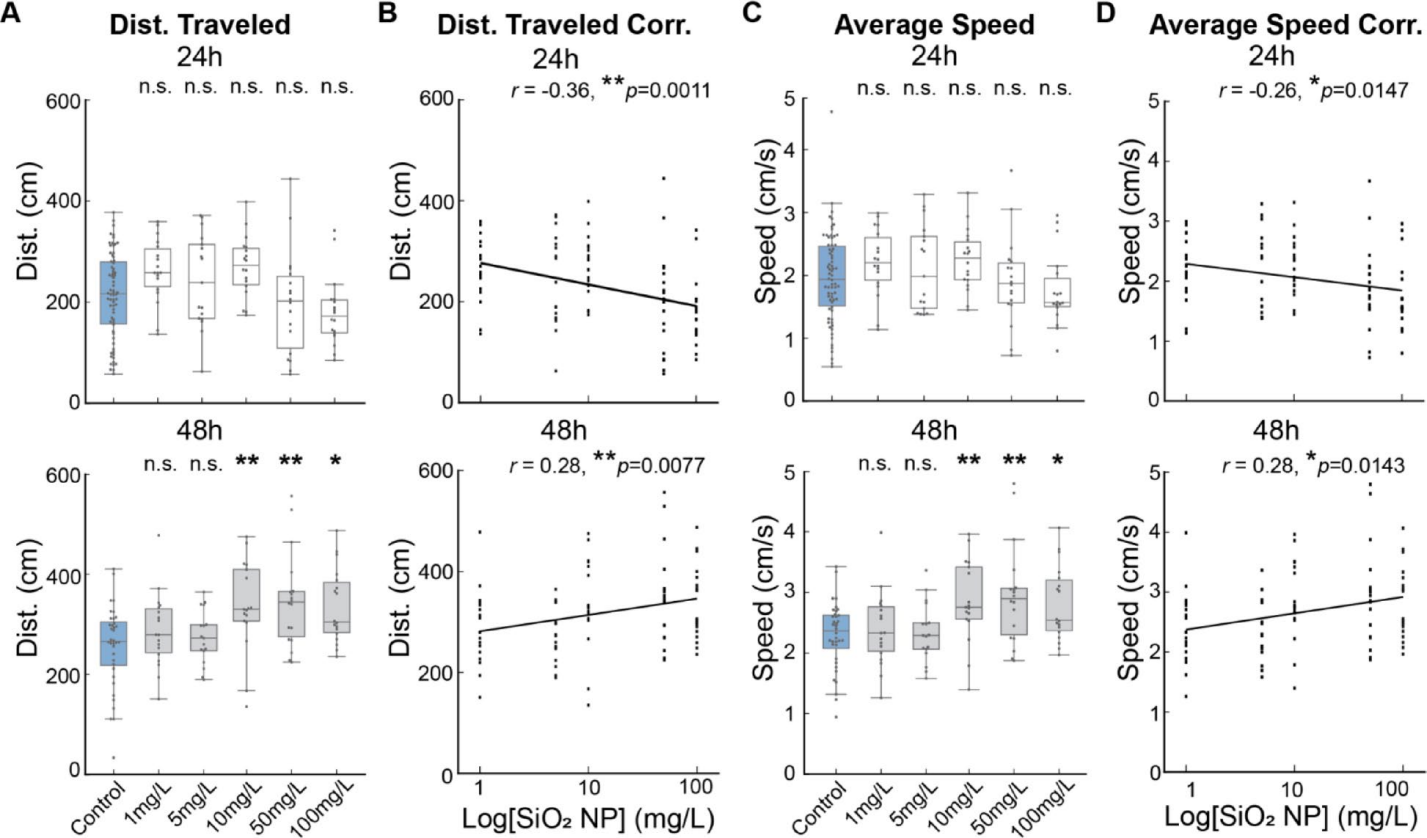
SiO_2_ NP alters locomotor activities in zebrafish larvae. (**A**) Distance (Dist.) traveled after exposure to concentrations of SiO_2_ NP. Top panel, the distances traveled by fish exposed for 24h differ significantly. **p = 0.0022, Kruskal-Wallis test. However, the distances traveled by fish exposed to SiO_2_ NP do not show significant differences compared to the control group. *p* > 0.05, Mann-Whitney U test. Bottom panel, the distance traveled by fish exposed for 48h differs significantly. ***p = 0.0007, Kruskal-Wallis test. Compared to the control, fish exposed to 10 mg/L and higher traveled significantly longer distances. **p = 0.0039 for 10 mg/L, **p = 0.0085 for 50 mg/L, *p = 0.0146 for 100 mg/L, one-tailed Mann-Whitney U test with Holm-Bonferroni correction. (**B**) Correlation (Corr.) between distance traveled and concentration. Top panel, the distance traveled by 24h exposed larvae negatively correlated with concentration. *r*=-0.36, **p = 0.0011. Bottom panel, the distance traveled by 48h exposed larvae positively correlated with concentration. *r* = 0.28, **p = 0.0077. (**C**) Average speed after exposure to concentrations of SiO_2_ NP. Top panel, the average speed swam by fish exposed for 24h differs significantly. ****p < 1E-6, Kruskal-Wallis test. Compared to the control, fish subjected to 10 mg/L and higher swam significantly faster. *p = 0.0162 for 10 mg/L, ***p = 0.0004 for 50 mg/L, ***p = 0.0004 for 100 mg/L, one-tailed Mann-Whitney U test with Holm-Bonferroni correction. Bottom panel, the average speed swam by fish exposed for 48h differs significantly. **p = 0.0013, Kruskal-Wallis test. Compared to the control, fish exposed to 10 and 100 mg/L swam significantly faster. **p = 0.0049 for 10 mg/L, **p = 0.0061 for 50 mg/L, *p = 0.0304 for 100 mg/L, one-tailed Mann-Whitney U test with Holm-Bonferroni correction. (**D **) Correlation between average speed and concentration. Top panel, the average speed swam by 24h exposed larvae negatively correlated with concentration. *r*=-0.26, *p = 0.0147. Bottom panel, the average speed swam by 48h exposed larvae positively correlated with concentration. *r* = 0.28, *p = 0.0143.

Next, we assessed the locomotor activities to identify the factors contributing to reduced exploration and increased ISI. In the 24h groups, traveled distances were significantly different among groups (**p = 0.0022, Kruskal-Wallis test), although pairwise comparisons between control and experimental groups were not significant (Fig. 5A, top panel; *p* > 0.05, Mann-Whitney U test). Furthermore, the distances traveled negatively correlated with the exposure concentration, demonstrating a concentration-dependent reduction in the locomotion (Fig. 5B, top; *r*=-0.36, **p = 0.0011). In contrast, larvae exposed for 48h at > 10 mg/L traveled significantly longer distances than the control group (Fig. 5A, bottom; ***p = 0.0007, Kruskal-Wallis test for group comparison; **p < 0.01 for 10 and 50 mg/L, *p = 0.0146 for 100 mg/L, one-tailed Mann-Whitney U test with Holm-Bonferroni correction). Strikingly, in contrast to the 24h groups, the total distance traveled by the 48 groups positively correlated with the concentration (Fig. 5B, bottom; *r* = 0.28, **p = 0.0077).

Furthermore, consistent with the distance traveled, the average speed (excluding the ISI periods, see methods for more details) displayed a similar pattern. The average speeds among 24h groups showed significant differences (****p < 1E-6, Kruskal-Wallis test), although pairwise comparisons between control and experimental groups were not significant (Fig. 5C, top; *p* > 0.05, Mann-Whitney U test). Furthermore, the average speed swam by 24h groups negatively correlated with the concentration (Fig. 5D, top; *r*=-0.26, *p = 0.0147). In contrast, larvae exposed for 48 showed significant difference in the average speed in multi-group comparisons (**p = 0.0013, Kruskal-Wallis test), with larvae exposed to > 10 mg/L swam significantly faster compared to the control (Fig. 5C, bottom; **p < 0.01 for 10 and 50 mg/L, *p = 0.0304 for 100 mg/L, one-tailed Mann-Whitney U test with Holm-Bonferroni correction). Additionally, swimming speed positively correlated with the concentration after 48h of exposure (Fig. 5D, bottom; *r* = 0.28, *p = 0.0143).

Taken together, these results further validated the dose-dependent effects of SiO_2_ NP exposure on locomotion and pointed to a dual effect associated with the exposure duration.

### SiO_2_ NP increases larvae trajectory angles

**Fig. 6:**
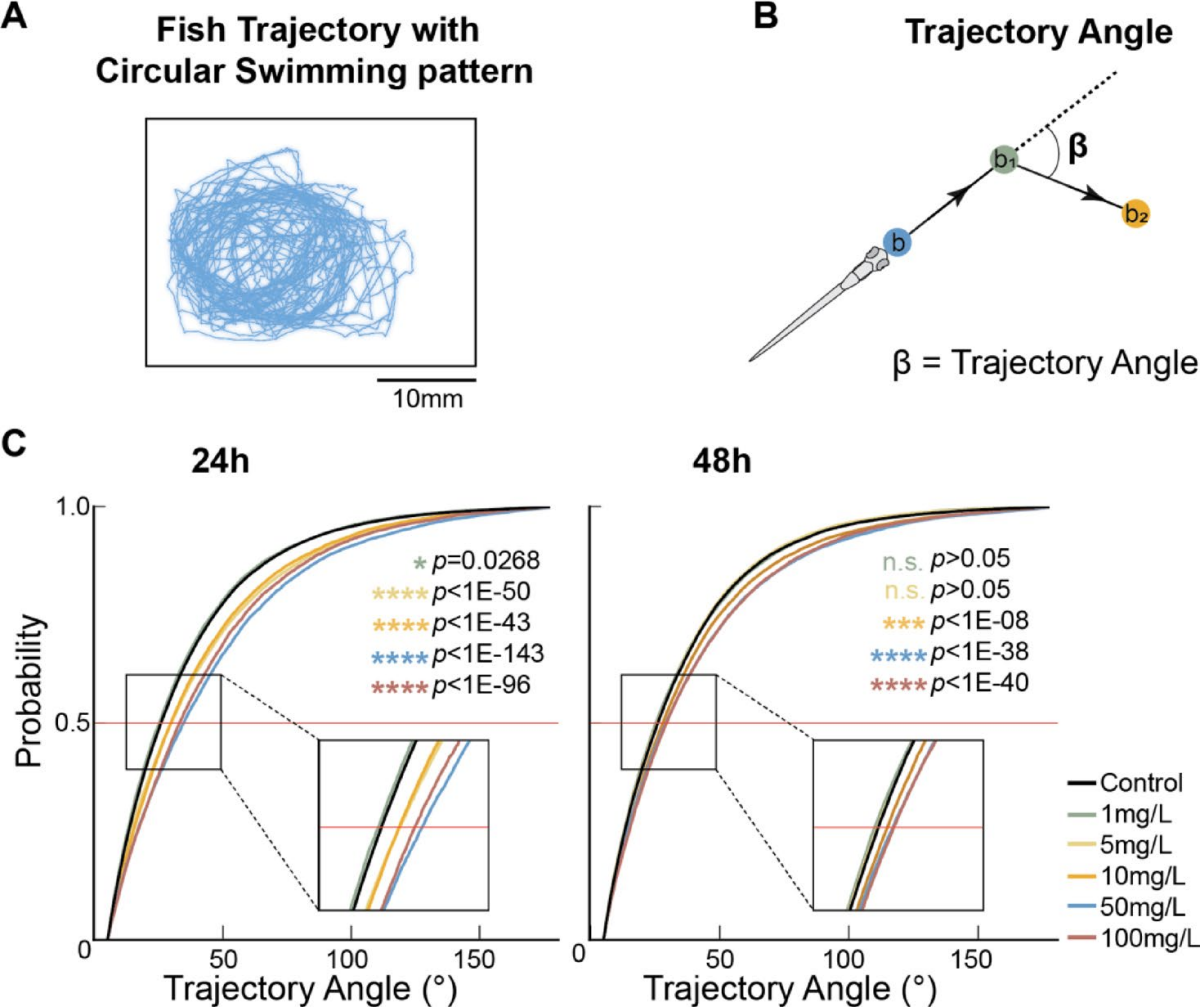
SiO_2_ NP increases larvae trajectory angle. (**A**) A sample SiO_2_ NP-treated fish trajectory demonstrating a circular swimming pattern. (**B**) The trajectory angle is defined as the degree difference in turning angle between every two consecutive swimming events, calculated with the b: initial position; b_1_: position after first bout; b_2_: position after second bout. (**C**) The cumulative distributions of trajectory angles. Left, compared to the control, the trajectory angles of fish exposed to 5 mg/L and higher for 24h exhibited larger turning degrees at the median (second quartile). *p = 0.0268 for 1 mg/L, ****p < 1E-50 for 5 mg/L, ****p < 1E = 43 for 10 mg/L, ****p < 1E-143 for 50 mg/L, ****p < 1E-96 for 100 mg/L, Kolmogorov–Smirnov test with Bonferroni correction. Right, compared to the control, the trajectory angles of fish exposed to 10 mg/L and higher for 48h exhibited larger turning degrees at the median (second quartile). ***p < 1E-08 for 10 mg/L, ****p < 1E-38 for 50 mg/L, ****p < 1E-40 for 100 mg/L, Kolmogorov–Smirnov test with Bonferroni correction.

Some fish’s trajectories displayed unusual circular swimming patterns (Fig. 6A), particularly larvae exposed to SiO_2_ NP. Thus, we hypothesize that larvae exposed to SiO_2_ NP exhibit a bias in motor orientation. To test this, we quantified their trajectory angles, i.e. the angle between every two consecutive swimming events (Fig. 6B).

Quantitation confirmed that SiO_2_ NP exposure for 24h increases their trajectory angles (Fig. 6C, left; *p = 0.0268 for 1 mg/L, ****p < 1E-43 for 5, 10, 50, and 100, Kolmogorov–Smirnov test with Bonferroni correction). Larvae exposed to 10 mg/L and higher for 48h also showed increases in the trajectory angle, i.e. a right shift in the distribution (Fig. 6C, right; ***p < 1E-08 for 10 mg/L, ****p < 1E-38 for 50 and 100 mg/L, Kolmogorov–Smirnov test with Bonferroni correction). This supports our hypothesis, where fish exposed to SiO_2_ NP were making turns at a greater angle, thus resulting in a circular trajectory path.

### Time-dependent effect of SiO_2_ NP in larvae

**Fig. 7:**
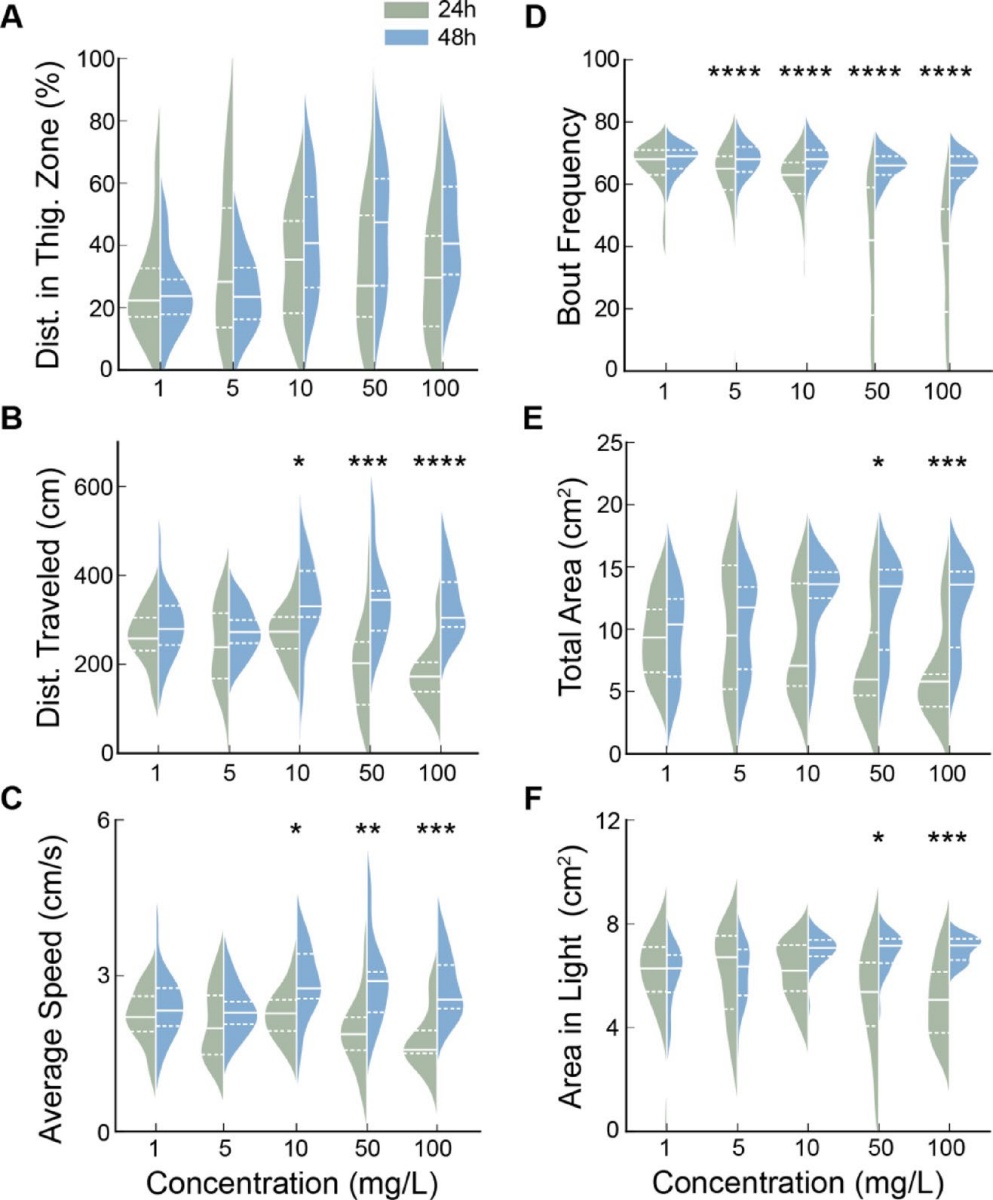
The effect of SiO_**2**_ NP is time-dependent. (**A**) The distance traveled in the thigmotaxis zone (Thig.). Fish exposed to SiO₂ NPs for 48h did not show a significant difference in anxiety-related behavior compared to those exposed for 24h at the same concentrations. *p* > 0.05, two-tailed Mann-Whitney U test with Bonferroni correction. (**B**) The total distance (Dist.) traveled. Fish exposed to 10 mg/L and higher for 48h traveled significantly longer distances than those exposed for 24h at the same concentrations. *p = 0.035 for 10 mg/L, ***p = 0.0008 for 50 mg/L, ****p < 0.0001 for 100 mg/L, two-tailed Mann-Whitney U test with Bonferroni correction. (**C**) The average speed. Fish exposed to 10 mg/L and higher for 48h swam significantly faster than those exposed for 24h at the same concentrations. *p = 0.0287 for 10 mg/L, **p = 0.0021 for 50 mg/L, ***p = 0.0002 for 100 mg/L, two-tailed Mann-Whitney U test with Bonferroni correction. (**D**) Bout frequency per minute. Fish exposed to 5 mg/L and higher for 48h exhibited more bout frequency than those exposed for 24h at the same concentration. ****p < 1E-9 for 5 mg/L, ****p < 1E-17 for 10 mg/L, ****p < 1E-70 for 50 mg/L, ****p < 1E-80 for 100 mg/L, two-tailed Mann-Whitney U test with Bonferroni correction. (**E**) The total area explored. Fish exposed to 50 mg/L and higher for 48h explored significantly larger areas than those exposed for 24h at the same concentration. *p = 0.0155 for 50 mg/L, ***p = 0.0009 for 100 mg/L, two-tailed Mann-Whitney U test with Bonferroni correction. (**F**)The area explored in the light side. Fish exposed to 50 mg/L and higher for 48h explored more area in the light side than those exposed for 24h at the same concentration. *p = 0.0171 for 50 mg/L, ***p = 0.0009 for 100 mg/L, two-tailed Mann-Whitney U test with Bonferroni correction.

Toxicity is usually associated with the exposure time^73^. We thus assessed whether the impacts of SiO_2_ NP on larval behavior were exposure time-sensitive. In terms of anxiety-related behaviors, we found that activity within the thigmotaxis zone did not differ between larvae exposed for 48h compared to those exposed for 24h (Fig. 7A; *p* > 0.05, two-tailed Mann-Whitney U test with Bonferroni correction), suggesting no anxiety-related differences.

For locomotion, larvae exposed to 10 mg/L and higher for 48h traveled significantly longer distances than those exposed for 24h at the same concentration (Fig. 7B; *p = 0.035 for 10 mg/L, ***p = 0.0008 for 50 mg/L, ****p < 0.0001 for 100 mg/L, two-tailed Mann-Whitney U test with Bonferroni correction). Moreover, 48h groups treated with 10, 50, and 100 mg/L exhibited significantly faster speed compared to 48h groups (Fig. 7C; *p = 0.0287 for 10 mg/L, **p = 0.0021 for 50 mg/L, ***p = 0.0002 for 100 mg/L, two-tailed Mann-Whitney U test with Bonferroni correction). These exposure time-dependent differences in locomotion were consistent with the observed dual effects of SiO_2_ NP. Furthermore, larvae exposed for 24h for concentrations of 5 mg/L and higher exhibited lower bout frequencies than those exposed for 48h (Fig. 7D; ****p < 1E-9 for 5, 10, 50, and 100 mg/L, two-tailed Mann-Whitney U test with Bonferroni correction), especially at higher concentrations of 50 mg/L and above.

Regarding the exploration, larvae exposed for 24h at 50 and 100 mg/L explored significantly less area within both the entire arena (Fig. 7E; *p = 0.0155 for 50 mg/L, ***p = 0.0009 for 100 mg/L, two-tailed Mann-Whitney U test with Bonferroni correction) and only the light side (Fig. 7F; *p = 0.0171 for 50 mg/L, ***p = 0.0009 for 100 mg/L, two-tailed Mann-Whitney U test with Bonferroni correction). These findings suggest that the duration of SiO_2_ NP exposure influences larval behaviors, generally resulting in a hyperactivating effect after 48h and a sedating effect after 24h.

## Discussion

As the application of nanoparticles progresses, highly prominent materials, such as, silica nanoparticles (SiO_2_ NPs) have been extensively used in the manufacturing and medical industry^74^. However, their potential for neurotoxicity remains inadequately understood. In this study, we have developed the zebrafish larvae behavioral model as a framework to investigate the impact of nanomedicines on brain functions. By applying this framework, we have demonstrated that SiO_2_ NP exposure can alter locomotion and elevate anxiety-related behavior in larval zebrafish before inducing apparent physical illness.

First, this work contributes to a solid foundation for future studies in dissecting of functional neural pathways underlying sensorimotor behaviors altered by NP exposures. Zebrafish at the late larval stage provide excellent optical access to visualize toxicology effects without disrupting development while still maintaining nearly transparency and relatively small brain and body size. These benefits make larval zebrafish ideal for non-invasive whole-brain neural recording at high speed and single-cell resolution to study underlying neural mechanisms. Hence, this study presents a framework developed using larval zebrafish that enabled early detection of nanomaterial neurotoxicity using SiO_2_ NP with low-dose exposure, providing a model for further neurological investigation.

Second, we demonstrated the dose-dependent effects of SiO_2_ NP on locomotion and anxiety. We used different concentrations (low: 1, 5 mg/L; high: 10, 50, 100 mg/L) and exposure durations (24h; 48h) of SiO_2_ NP and tested behavioral outcomes using light/dark preference assay. We observed dose-dependent elevations in anxiety and immobility (24h), as well as reductions in exploratory behaviors, while no alterations in phototaxis after exposure. These findings align with previous studies in mice and adult zebrafish where SiO_2_ NP exposure had an anxiogenic effect, including induced anxiety or depression-like behavior, as well as suppressive locomotive and exploratory behaviors^65,71,72,75^. We also found other dose-dependent behavioral phenotypes that have not been reported before, including extended inter-swim interval (ISI) and increased trajectory angles, respectively implying induced immobility and altered tail-bending amplitude. This adds another layer of evidence validating the dampened locomotion due to exposure, which guides future studies in neural mechanisms.

Third, our results suggest a potential role of the exposure duration in modulating SiO_2_ NP on behavior. We found that SiO_2_ NP has a biphasic effect on macro-scale locomotor activities (i.e. distance traveled and swimming speed), which were strikingly negatively correlated with the concentrations after 24h exposure but positively correlated with the concentration after 48h exposure. This biphasic effect parallels the effects observed with selective serotonin reuptake inhibitors (SSRIs) clinically and in animal models^76-78^, such as fluoxetine where acute exposure led to locomotion reduction while chronic exposure led to elevation in rats^79,80^. These observations suggest complexity of the effects and indicate that additional study is needed to understand the underlying mechanisms. Next, the exposure duration differences further validated the biphasic effects. While no difference in anxiety levels was found, the 48h groups exhibited higher locomotor activities compared to the 24h groups. This finding implies a possible time-sensitive effect associated with exposures to concentrations of 10 mg/L and higher.

Collectively, our work with SiO_2_ NP has led to the development of a promising behavioral framework that could be adopted for an array of future opportunities. First, further investigation into the biodistribution of NPs in larval zebrafish will visualize the localization of NPs which in turn offers toxicological insights. It will allow for the identification and modeling of neural pathways underlying the altered sensorimotor behaviors. Second, combining this behavioral assay with whole-brain calcium imaging will dissect the neural mechanism of sensorimotor transformation altered by nanomaterial neurotoxicity. Third, this framework is applicable for large-scale behavioral screening across a broad range of nanomaterials. It can be further extended to include other sensory stimuli, beyond the light/dark preference assay, which is primarily used to assess visual and anxiety-related behaviors in larvae^68^. This expansion will enable more refined assessment of other potential risks of nanomaterials linked with their chemical composition, and physical and chemical properties^81-83^. Future studies using this behavioral model in combination with whole-brain imaging could facilitate complete profiling of the neuromotor transformation mechanisms underlying nanomaterial neurotoxicity.

## Materials and methods

### Animals

All zebrafish experiments were carried out under the protocol (AUP 20013018) approved by the University of Toronto Local Animal Care Committee (LACC) and complied with the regulations of the Canadian Council on Animal Care (CCAC). The study was conducted following the ARRIVE guidelines. Wild-type zebrafish (*Danio rerio*) with a mixed AB and TL background lineage were used, obtained from Dr. Vincent Tropepe at the University of Toronto. The zebrafish used in this study for behavioral experiments were 16 days post-fertilization (dpf) and housed in a facility at 28 °C with a light cycle between 7:00 AM and 9:00 PM. Zebrafish were divided into two major groups by exposure duration: 24h (control, *n* = 70; 1 mg/L, *n* = 18; 5 mg/L, *n* = 17; 10 mg/L, *n* = 18; 50 mg/L, *n* = 18; 100 mg/L, *n* = 18); 48h (control, *n* = 36; 1 mg/L, *n* = 18; 5 mg/L, *n* = 17; 10 mg/L, *n* = 17; 50 mg/L, *n* = 18; 100 mg/L, *n* = 18).

### SiO_2_ NP acquisition and characterization

The silica nanoparticles (SiO_2_ NPs) were purchased from Sigma-Aldrich (St. Louis, MO) as nanopowder at ≥ 99.5% purity. The size of the NPs ranges from 10 to 20 nm and the diameter was confirmed by transmission electron microscopy (TEM, Hitachi HT7700) with an acceleration voltage of 80 kV. For the TEM grid preparation, 10 µl of particle solution was drop-cast on a carbon-coated copper grid and dried for 12 h. The size of 150 nanoparticles was determined to calculate the mean diameter and standard deviation. SiO_2_ NP size distribution was measured using ImageJ software. Silicon dioxide nanopowder was dispersed with Milli-Q H_2_O with no further purification and then diluted with fish water (salt concentration: 0.06 g/L) to concentrations of 1 mg/L, 5 mg/L, 10 mg/L, 50 mg/L, and 100 mg/L.

### SiO_2_ NP exposure

Zebrafish larvae were exposed to SiO_2_ NP through waterborne exposure at 14–15 dpf in conventional 250mL glass beakers, each containing 200mL of treatment solution. The exposure durations were set to either 16–23 h (24h) or 44–51 h (48h) to allow sufficient time for behavioural assays as NP effects can diminish if animal are removed too early. During 24h exposure, no food was administered to larvae to minimizing potential confounding effects from food, whereas during 48h exposure, they received one feeding to ensure consistent nutrient availability. The SiO_2_ NP solution was refreshed after the feeding to maintain optimal water quality as well as consistent concentration and dispersity of the SiO_2_ NPs.

### Light and dark preference behavioral assay

To phenotype behavioral alterations from SiO_2_ NP exposure, a light and dark preference assay was employed using a custom-designed behavioral apparatus. All experiments were conducted on 16 dpf. After exposure, larvae were transferred from the NP solution and introduced in the light zone of a custom-made square six-well chamber with black walls (10 cm in width and 1 cm in height) filled with fish water, with each well measuring 5 cm in length, 3.33 cm in width, and 0.6 cm in height, housing a single larva. The chamber walls were made in black to obscure the larvae’s visibility of each other. A projector (Yoton Y3 mini) and an achromatic doublet (Thorlabs, AC508-180-A-ML) were used to project light/dark stimuli onto the bottom of the arena, reflecting off a cold mirror (Edmund Optics, 64450). The environment of this behavioral apparatus was illuminated by a stripe of near-infrared (NIR) 950 nm light LEDs (Aliexpress). After an acclimation period of five minutes, larval behavior was collected under a camera (Blackfly S BFS-U3-23S3M, FLIR Systems) equipped with an 850 nm long pass filter (Thorlabs, FGL850) at 15 fps for 20 min.

### Behavior quantification

FIJI^67^ was used for video processing and behavioral tracking was performed offline with DeepLabCut™^84^. Custom-written Python scripts analyzed kinematic data, including metrics such as animal trajectories, zone preferences, explored areas, thigmotaxis behaviors, entry duration, inactive time, distance traveled, average swimming speeds, and trajectory turning angles.

Normalized activity density was calculated by adjusting the multivariate kernel density estimate (KDE) of the x- and y-coordinates, representing the larvae’s location during each trial, to a normalized scale from 0 to 1, and subsequently across all trials. This approach allowed for visualizing their preferred localizations on a heat map. It facilitated the comparison of the probability distributions of the fish’s presence across different two-dimensional areas. Thigmotaxis zones were designed as areas within 5 mm of any wall and the border between dark and light zones (Fig. 3B). The area explored by each larva was calculated using convex hull formations based on their localization. Average swimming speed was calculated using only swimming events as the distance traveled per second, excluding periods of inter-swim intervals (ISIs). The ISI was calculated as the duration between each swimming event using a threshold of 0.16 mm in distance, which is less than 5% of the animal’s body length. The trajectory angle is defined as the angle at which the fish turns (Fig. 6B), was calculated using every two consecutive swim bouts^85^. Swim bouts that occurred within 600 ms of a preceding or subsequent bout were excluded from the analysis to avoid potential cross-talk between bouts and angles less than five degrees were removed from the analysis^86^. Trajectory angle distributions were visualized using the Empirical Cumulative Distribution Function (ECDF).

### Statistical analysis

Wilcoxon signed-rank tests were used to analyze within-group behavior comparisons between the light and the dark zones. Kolmogorov-Smirnov tests were used to analyze ISI and trajectory angle distributions. Two-sided Mann-Whitney U tests were used to evaluate between-group analyses (control vs. treated group; 24h vs. 48h group) of various measured parameters. All of the statistical tests were adjusted using the Bonferroni correction method. Kruskal-Wallis H tests were conducted for multi-group comparisons. If Kruskal-Wallis H tests presented significance, the Holm-Bonferroni correction method was used to perform more powerful corrections. Correlations between behavioral measurements and logged concentrations were analyzed using linear regression.

## Electronic supplementary material

Below is the link to the electronic supplementary material.

Supplementary Material 1

Supplementary Material 2

## Data Availability

All datasets and supporting materials of the current study are available from the corresponding author upon reasonable request.
