# Supplementary material for: Altered locomotion and anxiety after exposure to SiO2 nanoparticles in larval zebrafish
Source: Sci Rep. 2025 May 25;15:18229. doi: 10.1038/s41598-025-02599-3 (PMC12104406; doi:10.1038/s41598-025-02599-3)
Supplement: Supplementary file 1 — Supplementary Material 1 [file 41598_2025_2599_MOESM1_ESM.pdf]

Supplementary Figures

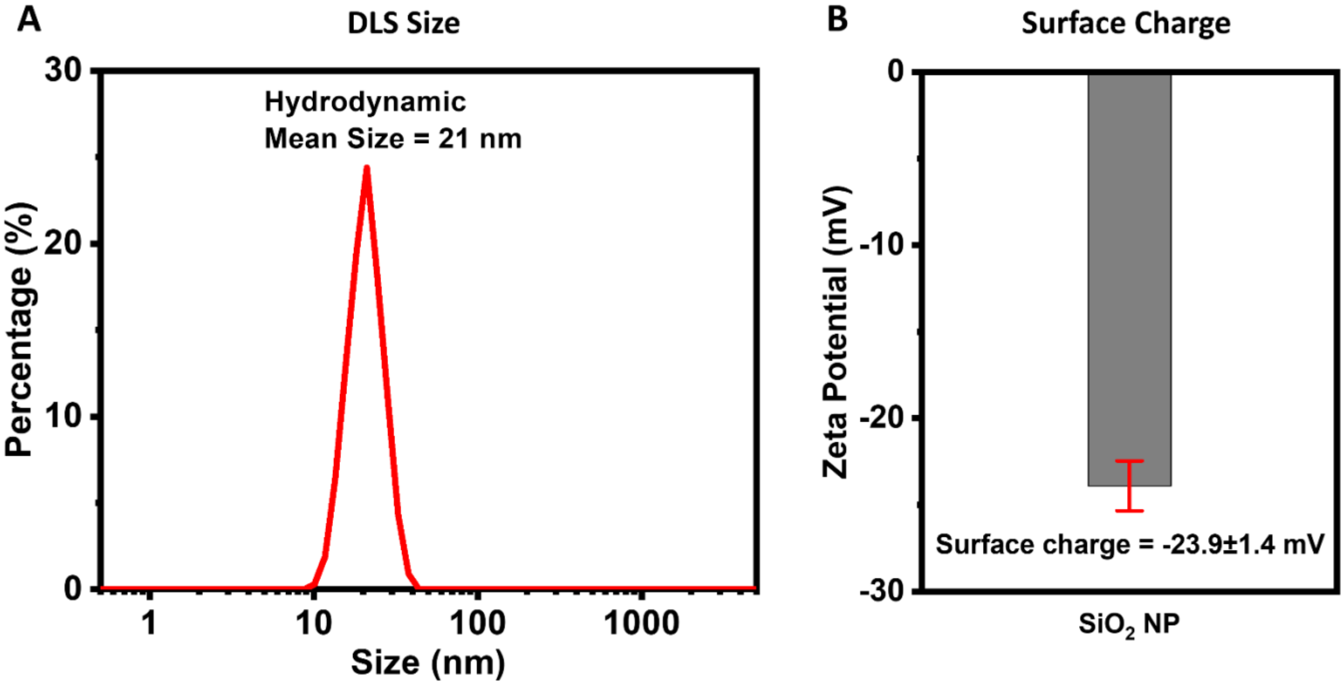

Figure S1. Characterization of silica nanoparticles.

(A) Hydrodynamic size distribution of silica nanoparticles measured using Dynamic Light Scattering (DLS). The mean hydrodynamic size is 21nm, reflecting monodistribution.

(B) Silica nanoparticles exhibit a negative surface charge, with a zeta potential of -23.9 ± 1.4mV.

Supplementary Video (*Video S1.mp4*)

Video S1. Alterations in exploration patterns after 24-hour exposure to SiO_2 NPs. Related to Figures 2 & 4. One example of fish from 3 different treatment groups are shown. Consistent with the quantified results in Figure 2, all fish exhibited robust light preference. Consistent with results in Figure 4, 24-hour SiO_2 NPs exposure led to suppressed exploratory behavior, with more pronounced effects observed at higher concentrations. Recording frame rate: 15 fps. Playback: 20 × real time.
